# Supplementary material for: Trans cohort metabolic reprogramming towards glutaminolysis in long-term successfully treated HIV-infection
Source: Commun Biol. 2022 Jan 11;5:27. doi: 10.1038/s42003-021-02985-3 (PMC8752762; doi:10.1038/s42003-021-02985-3)
Supplement: Supplementary file 2 — Supplementary Information [file 42003_2021_2985_MOESM2_ESM.pdf]

Trans cohort metabolic reprogramming towards glutaminolysis in long-term successfully treated HIV-Infection

Flora Mikaeloff[1], Sara Svensson-Akusjärvi[1], George Mondinde Ikomey[2.3], Shuba Krishnan[1], Maike Sperk[1], Soham Gupta[1], Gustavo Daniel Vega Magdaleno[4], Alejandra Escós[1], Emilia Lyonga[2,3], Marie Claire Okomo[2,3], Claude Tayou Tagne[3], Hemalatha Babu[5,6], Christian L Lorson[7,8], Ákos Végvári[9], Akhil C. Banerjea[10], Julianna Kele[11], Luke Elizabeth Hanna[5], Kamal Singh[7,8], João Pedro de Magalhães[4], Rui Benfeitas[12], Ujjwal Neogi[1,13]*

[1]The Systems Virology Lab, Division of Clinical Microbiology, Department of Laboratory Medicine, Karolinska Institute, ANA Futura, Campus Flemingsberg, Stockholm, Sweden.
[2]Center for the Study and Control of Communicable Diseases (CSCCD), Faculty of Medicine and Biomedical Sciences, University of Yaoundé 1, P.O. Box. 8445 Yaoundé, Cameroon
[3]Department of Microbiology, Haematology, Parasitology and Infectious Disease, Faculty of medicine and Biomedical Sciences, University of Yaoundé 1, Cameroon
[4]Integrative Genomics of Ageing Group, Institute of Life Course and Medical Sciences, University of Liverpool, Liverpool, UK
[5]Department of HIV/AIDS, National Institute for Research in Tuberculosis, ICMR, Chennai 600031, India
[6]Division of Microbiology and Immunology, Yerkes National Primate Research Center, Emory Vaccine Center, Emory University, Atlanta, GA 30329.
[7]Christopher S. Bond Life Sciences Center, University of Missouri, Columbia, MO, 65211, USA
[8]Department of Veterinary Pathobiology, University of Missouri, Columbia, MO, 65211, US
[9]Division of Chemistry I, Department of Medical Biochemistry and Biophysics, Karolinska Institutet, Stockholm, Sweden
[10]National Institute of Immunology, Aruna Asaf Ali Marg, New Delhi, India
[11]Department of Physiology and Pharmacology, Neurovascular Biology and Health, Karolinska Institutet, 171 77 Stockholm, Sweden
[12]National Bioinformatics Infrastructure Sweden (NBIS), Science for Life Laboratory, Department of Biochemistry and Biophysics, Stockholm University, S-10691 Stockholm, Sweden.
[13]Manipal Institute of Virology (MIV), Manipal Academy of Higher Education, Manipal, Karnataka, India

Running Head: Metabolic reprogramming in the long term HIV-infection

*Ujjwal Neogi, **Email:** ujjwal.neogi@ki.se

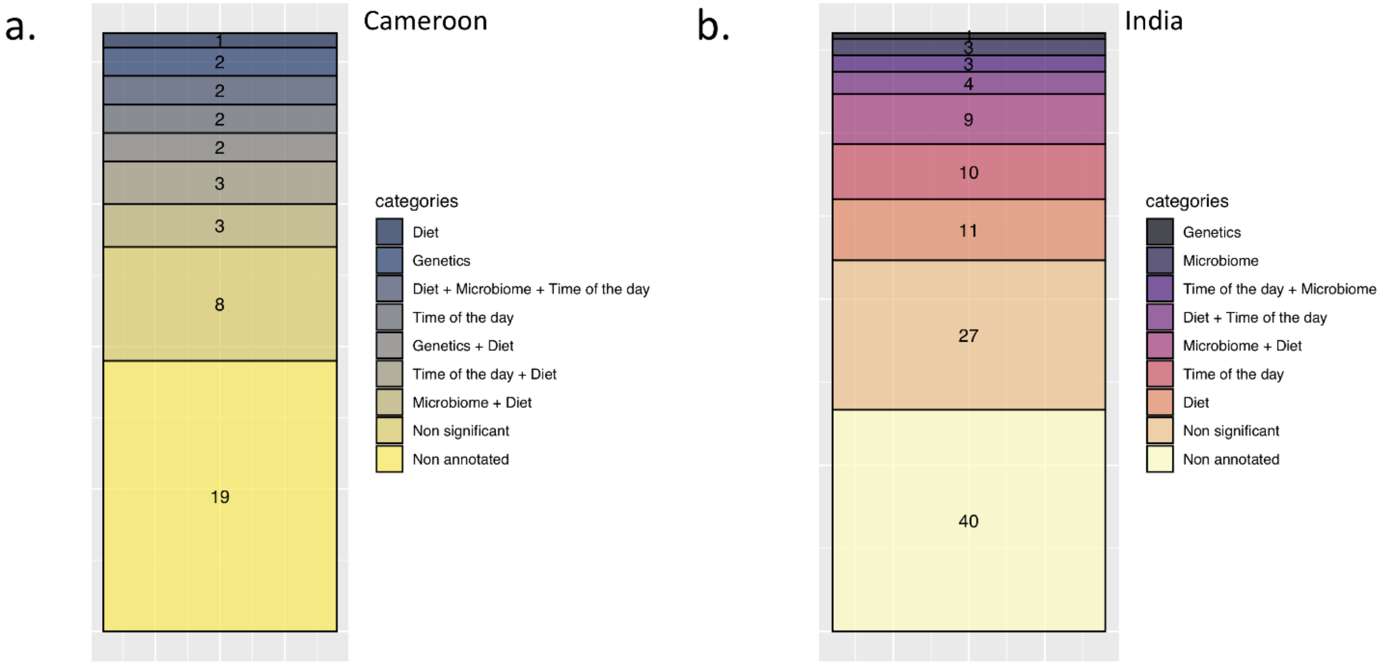

<u>Supplementary Fig 1</u>: Bar plot representing the number of detected metabolites associated significantly with each environmental category in Cameroon (a) and India (b) cohorts.

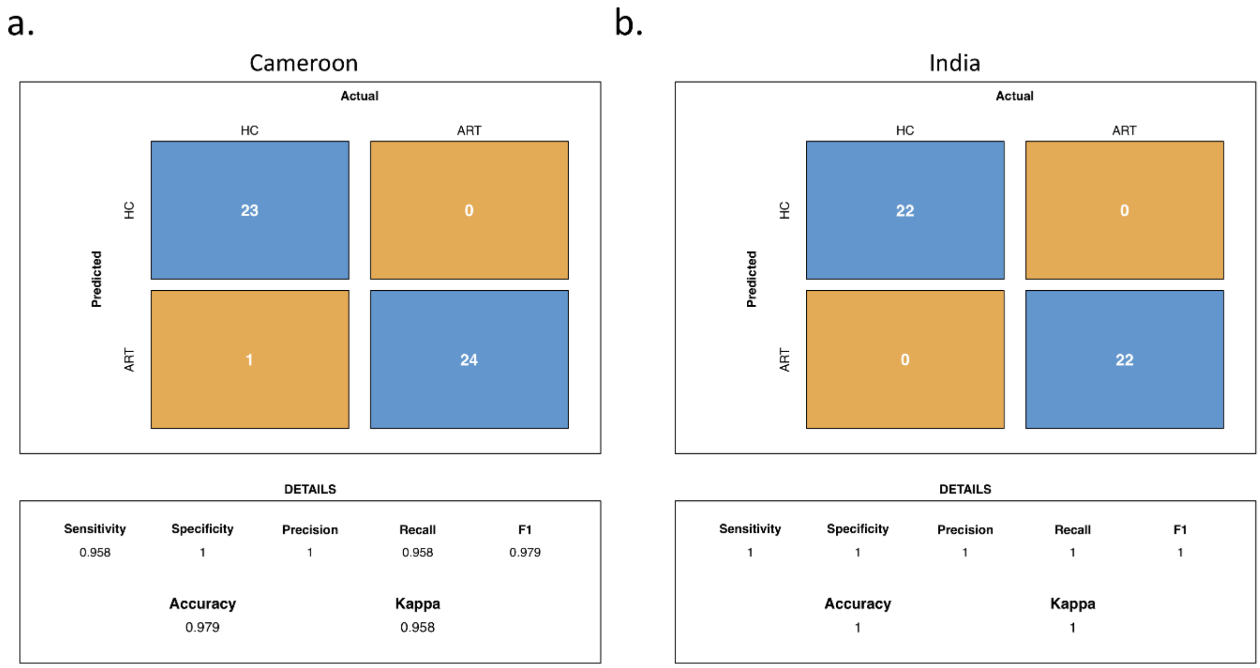

Supplementary Fig 2: Confusion matrices for Cameroon (a) and India (b) random forest models.

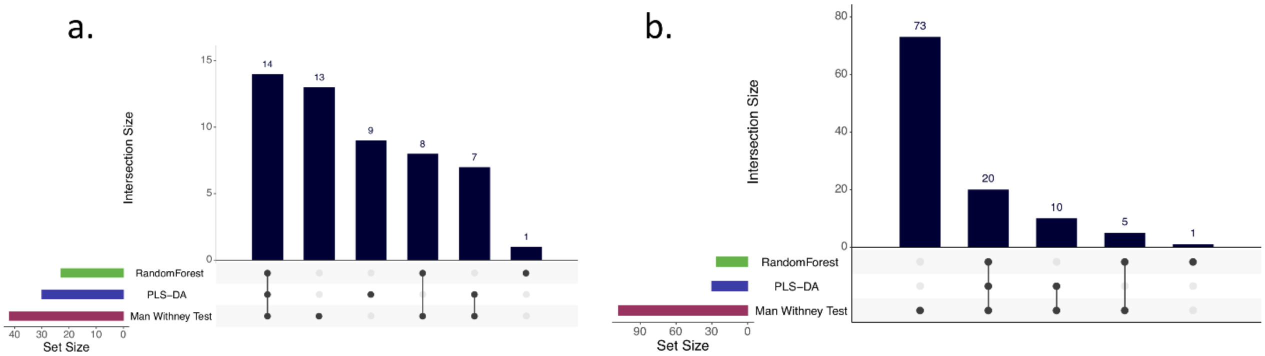

Supplementary Fig 3: Upset plot of metabolites with differential abundance between HC and ART patients identified by 3 different methods in Cameroon (a) and Indian (b) cohorts. Horizontal bars show the number of metabolites found with each method. Vertical bars display intersects between methods as indicated in the matrix below the graph.

a.

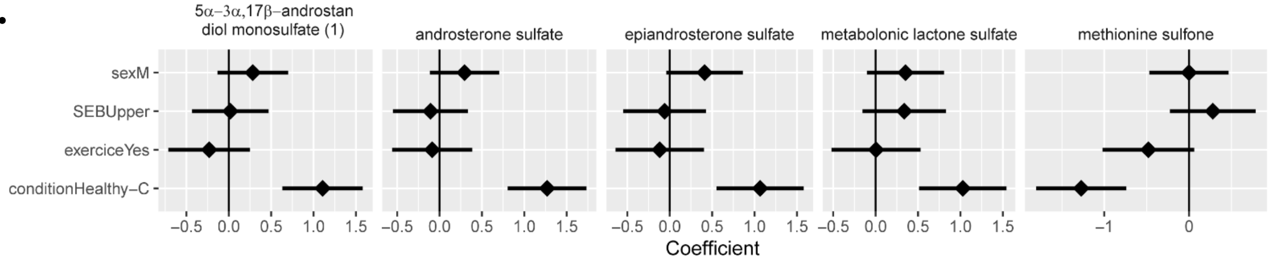

b.

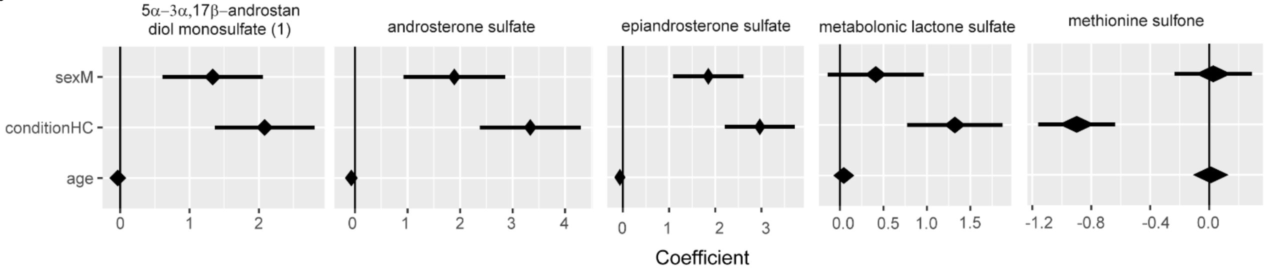

<u>Supplementary Fig 4</u>: Linear regression coefficients in Cameroon (a) and Indian (b) cohorts.

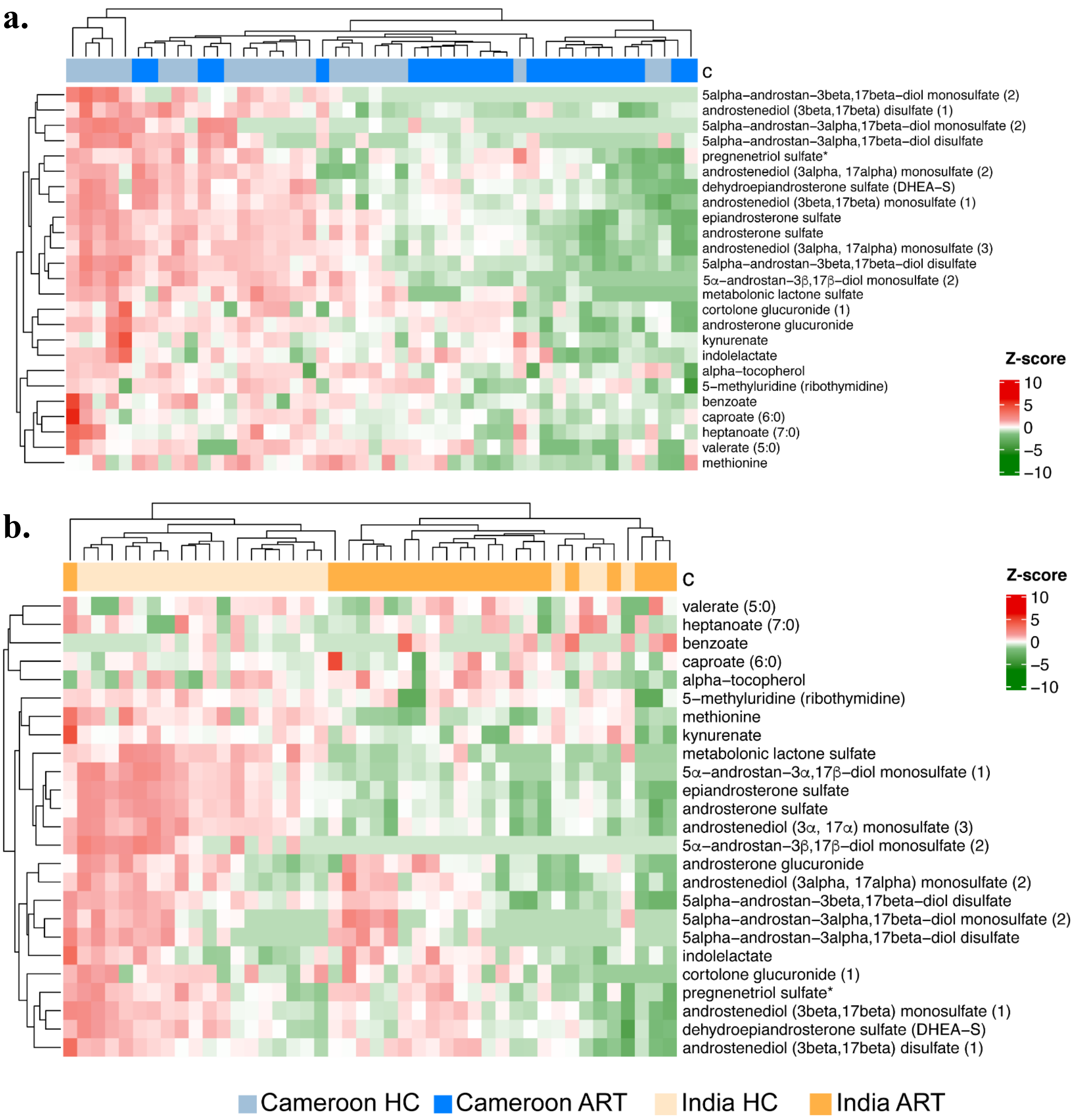

Supplementary Fig 5: Heatmaps of potential biomarkers and first neighbors in HC and ART in Cameroon (a) and Indian (b) cohorts. Data were log-transformed and z-score transformed.

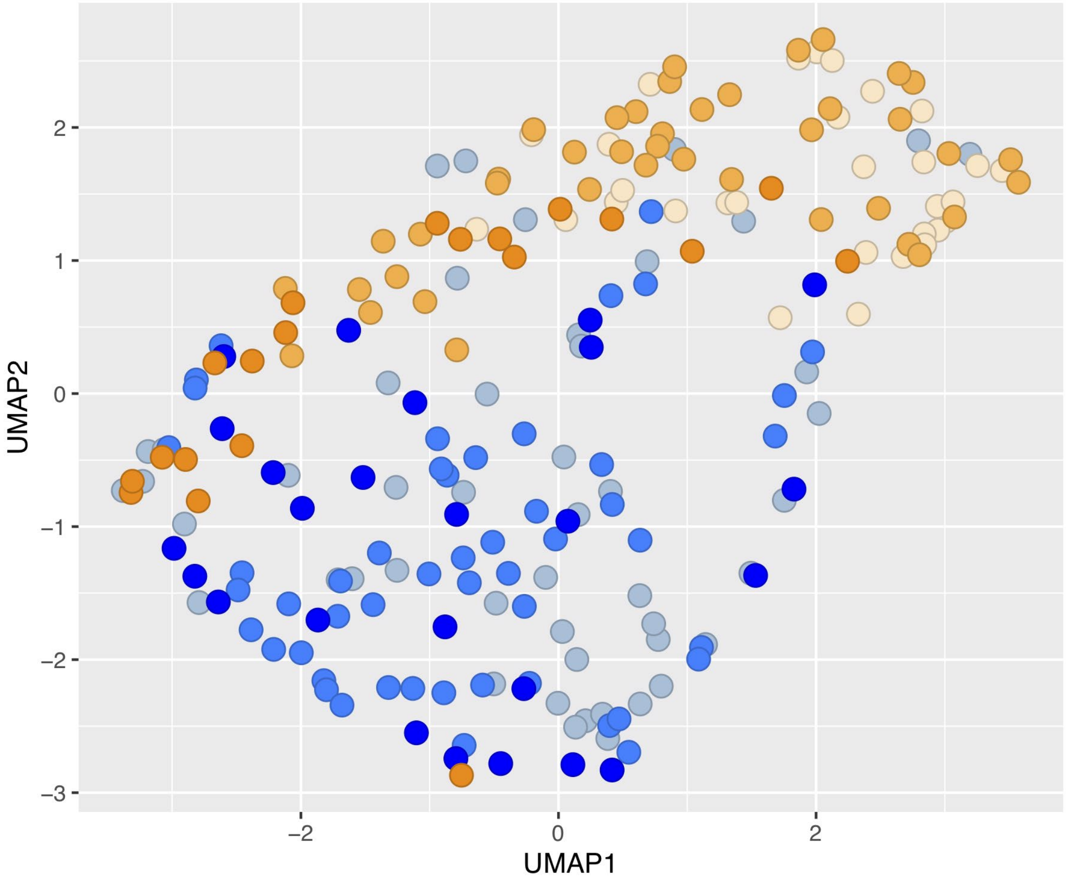

<u>Supplementary Fig 6</u>: UMAP visualization of AA (targeted metabolomics) for samples from Cameroon and Indian cohorts together. Cameroon HC = light blue; Camerron cART = steel blue; Cameroon naïve = blue, India HC = light orange; India cART = dark yellow, Indian naïve = Orange

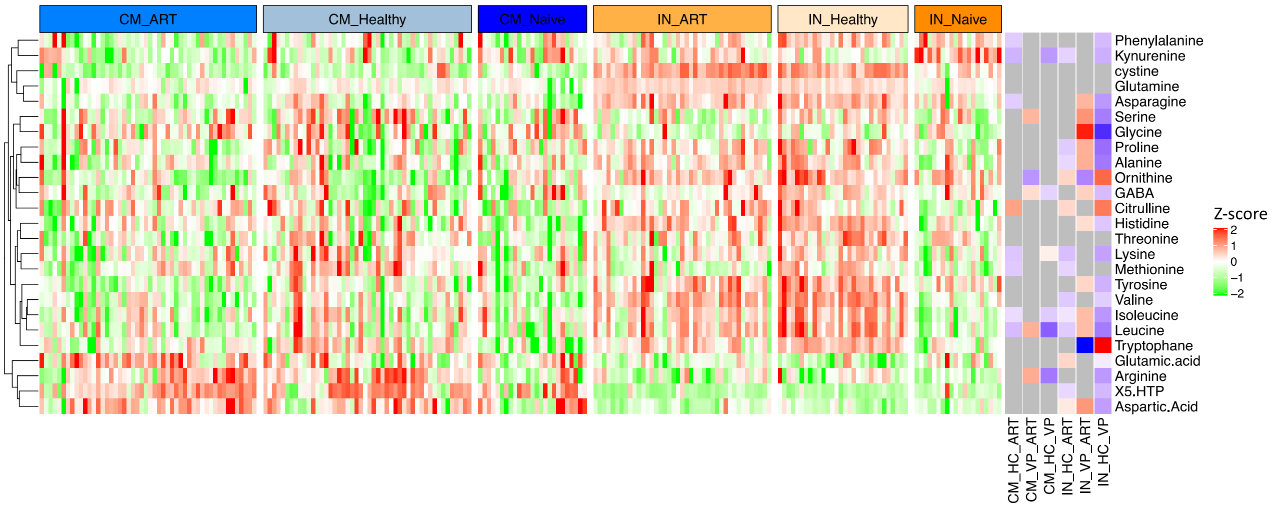

Supplementary Fig 7: Heatmap targeted metabolomics (amino acids) for Cameroon and India

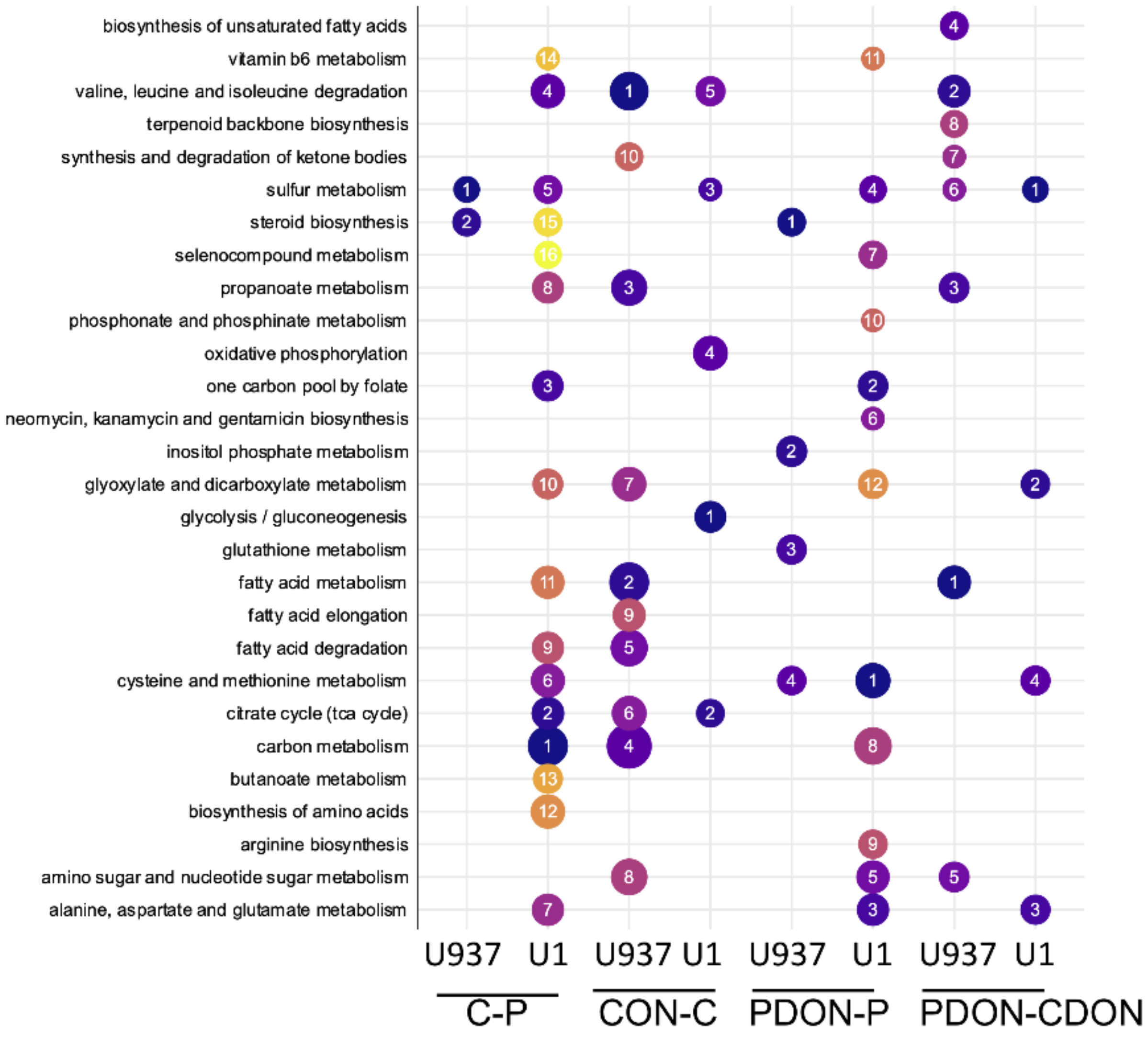

Supplementary Fig 8: Dot plot representing pathway analysis for all comparisons in U937 and U1 corrected for U937 cells. For each cell line, pathways for comparisons Control vs Prostatin (C-P), Control vs DON (CON-C), Prostratin vs Prostratin+DON (PDON-P), DON vs Prostratin+DON (PDON-CDON. Size of the bubble represents the number of proteins and the number, the rank of the pathway based on FDR.

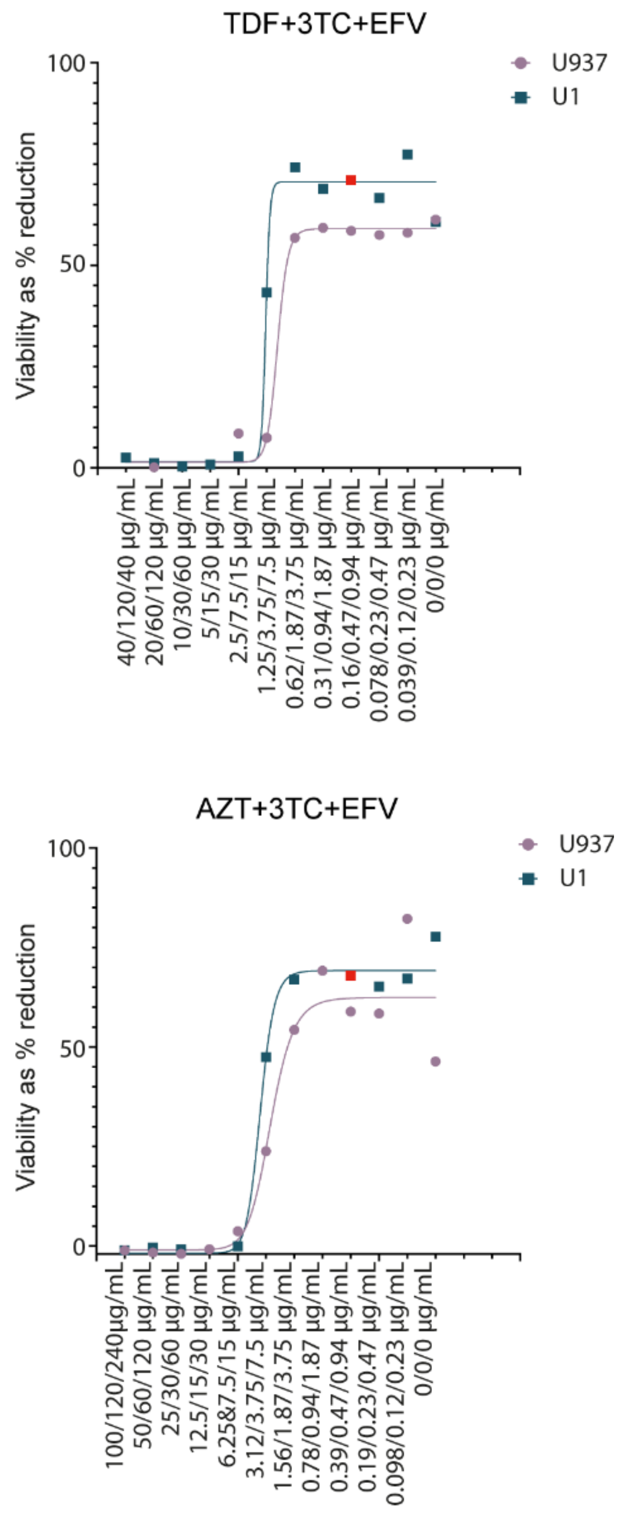

Supplementary Fig 9: Cytotoxicity of antiretroviral regimens TDF+3TC+EFV and AZT+3TC+EFV in monocytic cell lines, U937 (purple) and U1 (blue). The concentration of regimen used in Fig 5 is shown in red.

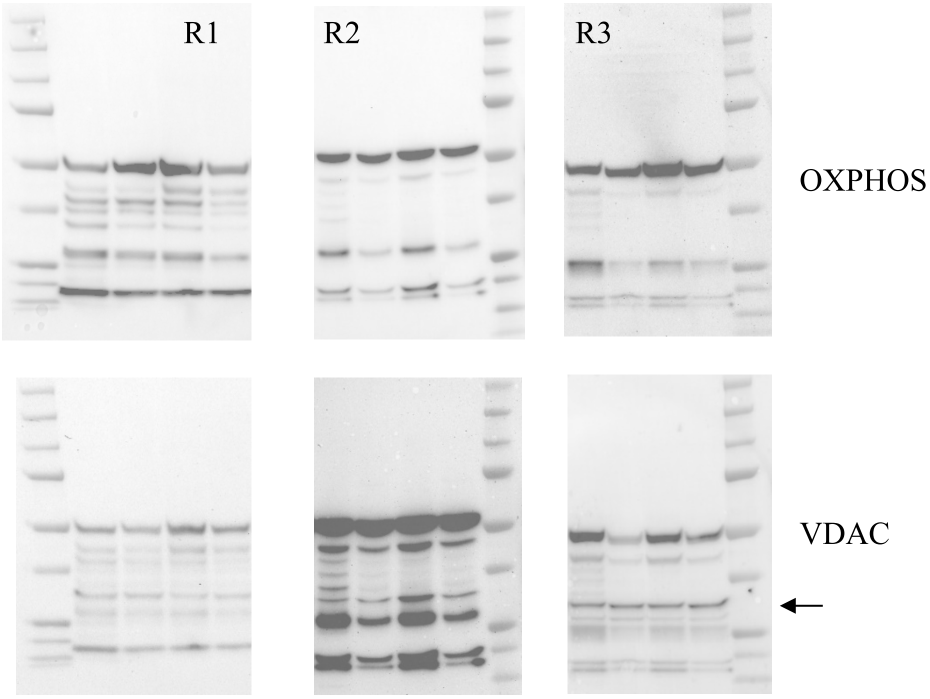

<u>Supplementary Fig 10</u>. Uncropped and unedited blot images

Supplementary Table 1. Demographic and clinical data for cohorts that were used for the targeted analysis.

	Cameroon				India				
	**control**	**PLWH on cART**	**Naive**	**p-value**	**control**	**PLWH on cART**	**Naive**	**p-value**	**Test**
**number**	50	50	25		30	41	20		
**Age in years, median (IQR)**	46.50 (40.00-53.00)	49.50 (40.50-56.75)	49.0 (40.0-56.0)	0.5828	46.00 (40.00-53.75)	45.00 (42.00-49.00)	43.00 (40.75-45.25)	0.242	Kruskal-Walis
**Gender, male, n (%)**	23 (46)	21 (42)	12 (48)	0.8645	13 (43.33)	25 (60.98)	9 (45)	0.2708	Chi square
**CD4 current, median (IQR)**	-	563.0 (464.2-793.5)	495.0 (382.0-558.0)	0.0031*	-	669.0 (465.0-779.0)	300.5 (213.2-527.0)	0.0004**	Mann-Whitney
**CD8, median (IQR)**	-	635.0 (386.0-798.8)	669 (385-900)	0.7065	-	796.5 (628.2-1,119.0)	1,278 (872-1,585)	0.0050*	t-test
**Viral load in copies/mL, median IQR)**	-	40.0 (40.0-40.0)	44,153 (21,412-215,343)	<0.001	-	149 (149-149)	55,700 (13,450-149,500)	<0.001	Mann-Whitney

Supplementary Method:

Targeted metabolomics of amino acids:

Standards and Calibration Curve

Amino acid standards (alanine, arginine, aspartic acid, cysteine, glutamic acid, glycine, histidine, isoleucine, leucine, lysine, methionine, phenylalanine, proline, serine, threonine, tyrosine, valine, glutamine, asparagine, GABA, citrulline, ornithine, taurine, tryptophan, 5-HTP, kynurenine and norvaline) were purchased from Sigma (St. Louis, MO, USA). Isotopically labeled amino acid standards (alanine ($^{13}C_3$, ^{15}N), arginine ($^{13}C_6$, $^{15}N_4$), aspartic acid ($^{13}C_4$, ^{15}N), cystine ($^{13}C_6$, $^{15}N_2$), glutamic acid ($^{13}C_5$, ^{15}N), glycine ($^{13}C_2$, ^{15}N), histidine ($^{13}C_6$, $^{15}N_3$), isoleucine ($^{13}C_6$, ^{15}N), leucine ($^{13}C_6$, ^{15}N), lysine ($^{13}C_6$, $^{15}N_2$), methionine ($^{13}C_5$, ^{15}N), phenylalanine ($^{13}C_9$, ^{15}N), proline ($^{13}C_5$, ^{15}N), serine ($^{13}C_3$, ^{15}N), threonine ($^{13}C_4$, ^{15}N), tyrosine ($^{13}C_9$, ^{15}N), valine ($^{13}C_5$, ^{15}N), Citrulline (d4), GABA ($^{13}C_4$), glutamine ($^{13}C_5$), asparagine($^{13}C_4$), ornithine (d6), tryptophan (d8), kynurenine (d4)) were obtained from Cambridge Isotope Laboratories (Andover, MA). Stock solutions of each compound were prepared at a concentration of 500 ng/µL and stored at -80ºC. A 10-point calibration curve (0.01-100 pmol/µL) was prepared by serial dilutions. 10µL of each calibration point was evaporated to dryness.

Mass spectrometry grade formic acid was purchased from Sigma-Aldrich (St Louis, MO, USA) and HPLC grade acetonitrile from Fisher Scientific (Fair Lawn, NJ, USA).

Extraction of amino acids in plasma

Amino acids were extracted by mixing 50 µL plasma with 450 µL 90:10 (v/v) Methanol: water solution containing norvaline at 2.2 pmol/µL as an internal standard. Each sample was extracted for 2 minutes using a mixer mill, incubated in the freezer for 2 hours and centrifuged at 4ºC, 14000 RPM, for 10 min. 25µL of the supernatant was transferred to micro vials and evaporated to dryness in a speed-vac concentrator. The samples were stored at -80 °C until analysis.

Amino acid derivatization with AccQ-Tag

Extracted samples were derivatized by AccQ-Tag™ (Waters, Milford, MA, USA) according to the manufacturers' instructions. Briefly, the dried extracts were resuspended in 20 µL of 20 mM HCl, and 60 µL of AccQ•Tag Ultra borate buffer spiked with all isotopically labelled internal standards at a final concentration of 0.833 pmol/µL was added to each sample. Finally 20 µL of freshly prepared AccQ•Tag derivatization solution was added and the samples were immediately vortexed for 10 seconds.

The dried calibration curves were prepared in a similar way using the same spiked Ultra borate buffer.

Samples were kept at room temperature for 30 minutes followed by 10 minutes at 55ºC. For each batch quality control samples and procedure, blanks were included.

Amino acids Quantification by LC-ESI-MSMS

Derivatized samples were analyzed using a 1290 Infinitely system from Agilent Technologies (Waldbronn, Germany), consisting G4220A binary pump, G1316C thermostated column compartment and G4226A autosampler with G1330B autosampler thermostat coupled to an Agilent 6460 triple quadrupole mass spectrometer equipped with a jet stream electrospray source operating in positive ion mode.

Separation was achieved injecting 1 µL of each sample onto a BEH C_{18} 2.1x100 mm, 1.7 µm column (Waters, Milford, MA, USA) held at 50ºC in a column oven. The gradient eluents used were H_2O 0.1% formic acid (A) and acetonitrile 0.1% formic acid (B) with a flow rate of 500 µL/min. The initial conditions consisted of 0% B, and the following gradient was used with linear increments: 0.54-3.50 minutes (0.1-9.1% B), 3.50-7.0 (9.1-17.0% B), 7.0-8.0 (17.0-19.70% B), 8.0-8.5 (19.7% B), 8.5-9.0 (19.7-21.2% B), 9.0-10.0 (21.2-59.6% B), 10.0-11.0 (59.6-95.0% B), 11.0-11.5 (95.0% B), 11.5-15.0 (0% B). From 13.0 minutes to 14.8 minutes the flow rate was set at 800 µL/min for a faster equilibration of the column.

The MS parameters were optimized for each compound as described in Supporting Information. MRM transitions for the derivatized amino acids were optimized using MassHunter MS Optimizer software (Agilent Technologies Inc., Santa Clara, CA, USA). The fragmentor voltage was set at 380 V, the cell accelerator voltage at 7 V and the collision energies from 14-45V, nitrogen was used as collision gas. The data was quantified using MassHunter™ Quantitation software B08.00 (Agilent Technologies Inc., Santa Clara, CA, USA) and the amount of each amino acid was calculated based on the calibration curves.

Mass Spectrometry parameters

Jet-stream gas temperature was 290°C with a gas flow of 11 L/min, sheath gas temperature 325°C, sheath gas flow of 12 L/min. The nebulizer pressure was set to 20 psi and the capillary voltage was set at 4 kV. The QqQ was run in Dynamic MRM Mode with 2 min retention time windows and 500 msec cycle scans.

Table 1. Retention times (rt), MRM-transition stages monitored (precursor ion and product ions) and collision energies of analyzed compounds.

Compounds	MRM transition		rt (min)	Collision Energy (V)
	Precursor Ion	Product Ion		
alanine	260.1	171	4.1	14
arginine	345.1	171	2.8	35
aspartic acid	304.1	171	3.4	18
cystine	581	171	5.2	25
glutamic acid	318.1	171	3.6	22
glycine	246.1	171	3.1	18
histidine	326.1	171	2.6	26
isoleucine	302.1	171	7.8	18
leucine	302.1	171	7.6	18

lysine	487.2	171	5.1	26
methionine	320.1	171	6.0	22
phenylalanine	336.1	171	8.1	18
proline	286.1	171	4.4	14
serine	276.1	171	3.1	14
threonine	290.1	171	3.7	18
tyrosine	352.1	171	5.7	18
valine	288.1	171	6.1	18
citrulline	346.2	171	3.4	30
GABA	274.1	171	4.1	18
glutamine	317.1	171	3.0	22
asparagine	303.1	171	2.8	18
ornithine	473.2	171	4.7	34
tryptophan	375.2	171	8.4	26
kynurenin	379.2	171	7.6	37
Internal standards				
Norvaline	288.1	171	6.4	18
alanine (13C3, 15N)	264.07	171	4.1	14
arginine (13C6, 15N4)	355.1	171	2.8	35
aspartic acid (13C4, 15N)	309.07	171	3.3	18
cystine (13C6, 15N2)	589	171	5.2	25
glutamic acid (13C5, 15N)	324.09	171	3.5	22
glycine (13C2, 15N)	249.05	171	3.1	18
histidine (13C6, 15N3)	335	171	2.6	26
isoleucine (13C6, 15N)	309.12	171	7.9	18
leucine (13C6, 15N)	309.12	171	7.6	18
lysine (13C6, 15N2)	495.1	171	5.2	26
methionine (13C5, 15N)	326.17	171	6.0	22
phenylalanine (13C9, 15N)	346	171	8.2	18
proline (13C5, 15N)	292.09	171	4.4	14
serine (13C3, 15N)	280.6	171	3.0	14
threonine (13C4, 15N)	295.08	171	3.7	18
tyrosine (13C9, 15N)	362.12	171	5.7	18
valine (13C5, 15N)	294.1	171	6.1	18
Citrulline (d4)	350.1	171	3.4	30
GABA (13C4)	278.1	171	4.0	18
glutamine (13C5)	322.1	171	3.1	22
asparagine(13C4)	307.1	171	2.8	18
ornithine (d6)	479	171	4.6	34
tryptophan (d8)	383.2	171	8.3	26
kynurenine (d4)	383	171	7.7	37
